# Recognition in a Social Symbiosis: Chemical Phenotypes and Nestmate Recognition Behaviors of Neotropical Parabiotic Ants

**DOI:** 10.1371/journal.pone.0056492

**Published:** 2013-02-22

**Authors:** Virginia J. Emery, Neil D. Tsutsui

**Affiliations:** Department of Environmental Science, Policy and Management, University of California, Berkeley, California, United States of America; Sheffield University, United States of America

## Abstract

Social organisms rank among the most abundant and ecologically dominant species on Earth, in part due to exclusive recognition systems that allow cooperators to be distinguished from exploiters. Exploiters, such as social parasites, manipulate their hosts’ recognition systems, whereas cooperators are expected to minimize interference with their partner’s recognition abilities. Despite our wealth of knowledge about recognition in single-species social nests, less is known of the recognition systems in multi-species nests, particularly involving cooperators. One uncommon type of nesting symbiosis, called *parabiosis*, involves two species of ants sharing a nest and foraging trails in ostensible cooperation. Here, we investigated recognition cues (cuticular hydrocarbons) and recognition behaviors in the parabiotic mixed-species ant nests of *Camponotus femoratus* and *Crematogaster levior* in North-Eastern Amazonia. We found two sympatric, cryptic *Cr. levior* chemotypes in the population, with one type in each parabiotic colony. Although they share a nest, very few hydrocarbons were shared between *Ca. femoratus* and either *Cr. levior* chemotype. The *Ca. femoratus* hydrocarbons were also unusually long–chained branched alkenes and dienes, compounds not commonly found amongst ants. Despite minimal overlap in hydrocarbon profile, there was evidence of potential interspecific nestmate recognition –*Cr. levior* ants were more aggressive toward *Ca. femoratus* non-nestmates than *Ca. femoratus* nestmates. In contrast to the prediction that sharing a nest could weaken conspecific recognition, each parabiotic species also maintains its own aggressive recognition behaviors to exclude conspecific non-nestmates. This suggests that, despite cohabitation, parabiotic ants maintain their own species-specific colony odors and recognition mechanisms. It is possible that such social symbioses are enabled by the two species each using their own separate recognition cues, and that interspecific nestmate recognition may enable this multi-species cooperative nesting.

## Introduction

Social organisms, ranging from microbes and insects to humans, dominate our planet. The success of any society is contingent on the ability to recognize members and non-members, and to maintain an efficient recognition system in the face of exploiters who might manipulate it [1]–[4]. Optimal social recognition systems minimize both rejection errors (that falsely reject members) and acceptance errors (that falsely accept non-members) by increasing the reliability of signals used in the recognition system. This can be done on the sender side, with more consistent relationships between cues and identity [5], [6], or on the receiver side by honing sensory perception and decision rules used by receivers to evaluate cues and assign identity [7]–[11].

For example, in a typical ant nestmate recognition system, the recognition cues are chemicals called cuticular hydrocarbons (CHCs), which can be both genetically and environmentally determined [12], [13]. A common nest odor, (the ‘gestalt odor’), is maintained through frequent social interactions, such as allogrooming, during which odors are exchanged among the interacting individuals. These interactions minimize recognition errors by homogenizing chemical cues across individuals [14]–[17]. Perceptually, both sensory habituation [18] and learning [19] allow ants to familiarize themselves with the gestalt odor and form a neural template of expected nestmate phenotypes. Ant nestmate recognition systems are reliable because of the frequent mixing of recognition cues, and the constant updating of individual’s neural templates as colony odors shift [4].

Social parasites gain entry to a host nest by manipulating or circumventing the recognition process, thus gaining access to the host’s social benefits, such as protection or brood care, to the detriment of the host species. In the ants, social parasites have evolved many times, with 230 described socially parasitic species, potentially representing up to 2% of total ant diversity [20]. Chemical mimicry or camouflage are the most commonly used methods of social integration. For example, the slave-making ants *Protomagnathus americanus* have locally adapted to increase their chemical similarity to their sympatric *Temnothorax* hosts [21]. Most ant social parasites gain entrance to their hosts’ nests by targeting closely related species and placing their brood in the same chamber as the host brood, producing a ‘mixed nest’ [22] which facilitates the chemical integration of the parasite into the host society [2]. However, some social parasites form ‘compound nests’ with their hosts, in which brood are kept in separate locations [20]. In these cases, called xenobioses, the two nest-sharing species are often distantly related, but still obtain a similar, shared colony odor [23].

In theory, however, cue mimicry is not absolutely necessary, and social integration can be achieved by other mechanisms [24]. For example, the perceptual component of recognition is not completely self-referent, as it can be expanded to include other species’ cues [19], [25]–[27]. This template broadening may reduce the host’s own conspecific recognition abilities, which can be a major cost of being parasitized [4]. Parasites can also escape detection by becoming imperceptible to their hosts, by either decreasing the amount of CHCs produced, or changing the type of compound expressed [28]. However, this ‘chemical insignificance’ could also reduce the ability of the parasite to discriminate conspecifics (a cost to the parasite) [4]. The altered recognition systems in socially parasitized nests can therefore be costly to both the host and parasite species.

In some cases, however, different species of ants can coexist in a single nest without any apparent parasitism [20]. This rare relationship is called parabiosis, and is known from fewer than 20 species, many in the genera *Camponotus* and *Crematogaster*. In parabiosis, two distantly related species, often of different subfamilies, share a nest and foraging trails, but keep brood separate in a compound nest [29]–[31]. Superficially, these nests resemble xenobiotic parasitism, but the parabiotic partners are thought to coexist in a mutualism, with both species benefitting from the nesting association. This has been measured by quantifying the contribution of each species to foraging, nest defense, and third party mutualisms, such as with plants or honeydew producers [32]–[35]. However, one unmeasured cost of the parabiotic relationship could arise from a compromised recognition system.

Within the ‘compound nests’ there have been very few investigations of recognition (summarized in Table 1). Due to the limited number of studies, it is unclear which features of the recognition systems differ in parabiotic (mutalistic) and xenobiotic (parasitic) nests, but there are a few trends. The parabiotic ants seem to share fewer chemical cues with each other than xenobiotic ants [23], [36], [37]. Parabiotic associations may allow for the development of heterospecific nestmate level recognition [25], or chemotype level recognition [38], whereas xenobiotic associations have not shown this specificity. It is also unclear whether the parabiotic association has impacted conspecific recognition, which is reduced in the host species of xenobiotic nests [36], [39]. There are also differences between different parabiotic systems. For example, in the genus *Camponotus*, species that live in parabiosis or who are tolerated by other species have unusually long-chained hydrocarbons that are mostly branched alkenes and dienes [40]. The facultatively parabiotic ant *Odontomachus mayi*, does not have these specialized hydrocarbons [25].

**Table 1:** Summary of published work on chemical phenotypes, and heterospecific and conspecific nestmate recognition behaviors in naturally occurring parabiotic and xenobiotic compound nests.

	Species	Cues shared between species? How many/total?	Range of HC chain lengths	Aggression to heterospecificnon-nestmates?	Aggression to conspecificnon-nestmates?	References
**Parabiosis (compound nests in possible mutualism)**						
1	*Camponotus femoratus*	Few (2/8)	37–45	No	Yes	The current study:
	*Crematogaster levior Type A* *Crematogaster levior Type B*	None (0/16)Few (2/15)	25–3329–41	Yes	Yes	Emery and Tsutsui 2012
2	*Camponotus rufifemur* black*Camponotus rufifemur* red	Few (3/46)Few (2/17)	21–4924–41	No	Yes	Menzel et al. 2008, 2009 [38], [53]
	*Crematogaster modigliani*	Few (5/28)	35–40	Yes, but only toforeign chemotype	Yes	
3	*Odontomachus mayi*	Few		Yes	Yes	Orivel et al. 1997 [25]
	*Crematogaster carinata*	Few		Yes	Yes	
**Xenobiosis (compound nests in likely parasitism)**						
4	*Solenopsis gayi*	Yes (14/21)	23–28	No	No	Errard et al. 2003 [54]
	*Camponotus morosus*	Yes (15/36)	23–31	No	Yes	
5	*Formicoxenus provancheri*	Yes (60/60)	21–37	No	Low	Lenoir et al. 1997 [23]
	*Myrmica incompleta*	Yes (60/65 )	21–37	No	Yes	Errard et al. 1992 [39]
6	*Formicoxenus quebecensis*	Yes (38/40)	23–31			Lenoir et al. 1997 [23]
	*Myrmica alaskensis*	Yes (38/62)	21–37			
7	*Formicoxenus nitidulus*	Yes (17/24, 19/28)	25–35	No	No	Martin et al. 2007 [36]
	*Formica rufa* *Formica lugubris*	Yes (14/22)Yes (19/35)	23–3523–33	No	Yes	
8	*Pseudomyrmex ferrugineus*	Yes (8/8, 81.8%)	∼25–31	No		Espelie et al. 1988 [37]
	*Parachartegus aztecus*	Yes (8/8, 94.3%)	∼25–31	No		

Here, we examined the recognition system of the parabiotic association between *Camponotus femoratus* (subfamily: Formicinae) and *Crematogaster levior* (*Cr. limata* spp. group, subfamily: Myrmicinae). These ants co-occur in parabiotic ant-gardens in the Amazon region of South America [41]–[43]. We assessed nestmate recognition systems in these parabiotic nests by examining the cuticular hydrocarbon cues of ants and the aggressive rejection of non-nestmates in pair-wise behavioral assays. By combining an investigation of con- and heterospecific recognition, we tested the hypothesis that parabiosis can lead to heterospecific nestmate recognition [25], and the hypothesis that the ants in these mixed species nests may have compromised conspecific recognition systems through template broadening [4]. Specifically, we ask: 1) Do parabiotic ants share cuticular hydrocarbon cues? 2) Is there evidence of heterospecific recognition? 3) Is there evidence of altered conspecific recognition, such as reduced aggression to conspecific non-nestmates?

Our investigation is only the second study to look at recognition in a common and obligate social symbiosis (the first being in SE Asia [38], [44]), and contributes to identifying features that distinguish non-parasitized from parasitized recognition systems. We find that in this parabiosis, both species maintain their own species-specific odors and conspecific recognition behaviours. We also find some evidence that ants may be able to distinguish between their heterospecific nestmates and non-nestmates. These recognition patterns are consistent with the hypothesis that these social symbioses are different than social parasitisms, and may be true inter-society mutualisms.

## Methods

### Study Site

Parabiotic nests of *Ca. femoratus* and *Cr. levior* ants were observed in the lowland Amazonian rainforest of French Guiana, near the village of Kaw (3° 30′ 43′′ N, 30° 15′ 54′′ W) in March 2010 and July 2010. All research conformed to the policies for field work and collection in that country, and no specific permits were required for the described field studies. None of the species collected for this study are listed as endangered or protected, and the study location is not privately-owned or protected in any way. Twenty colonies were selected, and a single accessible nest from each polydomous colony (colonies span several individual nest units) was used as a source of ants for the behavioral observations and chemical extractions. All chosen nests were separated by 100 m or more of nest-free space, and assumed to belong to different colonies because these polydomous colonies have clustered nests, and no ants were observed walking between the chosen nest pairs. The 20 selected colonies were haphazardly assigned to 10 independent colony pair comparisons. The location of each nest was recorded using GPS.

### Cuticular Hydrocarbon Extraction

For each nest (n = 20) we collected a pooled single-species sample of 3–5 ants for *Ca. femoratus* and 20–30 ants for *Cr. levior*, because *Cr. levior* workers are individually much smaller (2–3 mm) than *Ca. femoratus* workers (>1 cm). Each group of ants was freeze-killed and submerged in 10–200 µL of hexane for 10 minutes. The ants were removed and stored in 95% EtOH, and the hexane was evaporated for transport back to UC Berkeley. Each CHC sample was re-eluted in 200 µL of hexane, and filtered through a 1 cm hexane-rinsed silica column to separate polar and non-polar compounds. To maximize sample recovery, each column was further rinsed with 300 µL of hexane. The 500 µL sample containing the non-polar hydrocarbons was blown down under nitrogen gas to a 60 µL volume, of which 2 µL were injected and analyzed.

### Cuticular Hydrocarbon Extract Processing

Extracts were analyzed using electron impact-mass spectrometry (70 eV) on an Agilent 5975 C mass selective detector interfaced to an Agilent 7890A gas chromatograph fitted with an DB-5 column (30-m×0.32-mm i.d., Agilent Technologies). Two µL of each sample were injected at 325°C in splitless mode using helium as a carrier gas, with a flow rate of 54.8 mL/min, and the following temperature program: 100°C hold for 1 min, ramp of 15°C /min to 200°C, and then a 2nd ramp of 2°C /min to 325°C with a hold at 325°C for 10 min, for a total run time of 80.167 minutes. Each resulting chromatogram was first automatically integrated using Chemstation vE.02.00 (Agilent Technologies), and then manually integrated using ACDC Labs (Advanced Chemistry Development) to ensure consistent integration of smaller peaks. The identity of each compound was verified using both library comparisons and also by manual comparison of the mass spectra diagnostic ions and calculation of Kovats indices [45].

### Behavioral Observations

Approximately 50 ants of each species were collected directly from their nests using an aspirator and kept separate from the other species in vials (*Cr. levior*) or Fluon-coated boxes (*Ca. femoratus*). Only actively moving and undamaged ants were used in assays. All behavioral assays were 1 to 1 individual interactions in neutral arenas; we used small (5 cm×5 cm) covered petri dishes for the *Cr. levior* x *Cr. levior* and the *Cr. levior* x *Ca. femoratus* assays, and 15 cm×15 cm Fluon coated glass bowls for the *Ca. femoratus* x *Ca. femoratus* assays. Each assay dish was cleaned with soapy water and hexane, and air dried between trials to remove any chemical cues from previous ants.

All observations were for 3 minutes, and were only used in analysis if both ants made antennal contact with the other ant. Assays were performed blind to the source colony of the interacting ants. All interactions and their approximate duration were noted by transcribing observations of the following behaviors: presence/absence of trophallaxis, mandible flares, biting, spatulate sting extrusion (*Crematogaster*), defensive spraying (*Camponotus*), prolonged fighting, antennal boxing, and active running away. An overall behavioral score was assigned at the time of observation (0 = amicable, 1 = neutral, 2 = mandible flare, 3 = biting, 4 = sting extrusion or spraying, 5 = prolonged fighting). A second observer verified the transcribed interactions by watching a subset of the same interactions, and by reading all of the transcribed interactions and assigning an independent aggression score. Any inconsistent observations (ie: when the two observers were not in agreement) were excluded from the analysis (n = 33).

### Colony Combinations and Behavioral Pairings

We did both nestmate (two ants from the same nest) and non-nesmate (each ant from a different nest) comparisons, and both conspecific (*Cr. levior x Cr. levior* n = 211, and *Ca. femoratus x Ca. femoratus n = 214*), and heterospecific comparisons (*Cr. levior x Ca. femoratus, n = 188*) for both the nestmate and non-nestmate pairings. We aimed for a minimum of 60 assays for each colony pairing with 10 nestmate and 10 non-nestmate assays for each species combination. For the non-nestmate *Cr. levior x Ca. femoratus* comparisons, we did 5 comparisons of each type (ie: five comparisons with *Cr. levior* nest 1× *Ca. femoratus* nest 2, and five with *Cr. levior* nest 2× *Ca. femoratus* nest 1). The final dataset consisted of a total of 613 observations.

### Statistical Analysis for Chemical Data

All chromatogram peaks eluting after a retention time of 15 minutes (>C20 backbone length) were included in the analysis. We included only compounds with >1% total abundance for at least one colony, but noted ‘trace’ compounds found in amounts <1% of the total profile for all colonies. Cross-chromatogram peak identity was confirmed by comparing retention times and the mass spectra. Both the presence/absence of peaks and the relative proportion of each peak within a chromatogram were used for analysis. First, using the presence/absence data for all peaks, we compared the profiles using principle component analysis (PCA). Next, we compared the relative proportion data for all peaks of the same pooled profiles using nonmetric multidimensional scaling (NMDS). Since results for both analyses were similar, only the NMDS results are shown in the figures.

### Statistical Analysis for Behavioral Data

For our analysis, we used the presence/absence of aggression as our categorical response variable, using both a definition of aggression as any score 2–5, and a more conservative measure of aggression (presence of aggression only for scores 3–5). We used both measures because a behavioral score of 2 corresponds only to ‘mandible flare’, which is more ambiguous than biting (score of 3) or stinging (score of 4). The results were always comparable, so we are only presenting results from a definition of aggression as 2–5, but other analyses (with aggression scores 3–5) can be found in Table S1. For all assays if one ant showed aggression, we considered there to be ‘presence of aggression’ in that interaction. However, for the *Cr. levior x Ca. femoratus* interactions, we were able to determine whether one or both ants showed aggression, so we also analyzed the behavior of each species separately for the heterospecific assays.

We used generalized linear mixed models (GLMMs) with a binomial distribution and a logit link function with observation category (nestmates vs non-nestmate) as a fixed effect and chemotype combination (within vs between chemotype), and colony pair combination (#1–10) as random effects. We used likelihood ratio tests with reduced models to assess effect significances. Since there was an effect of colony pair number in some subsets of the data, indicating that certain colony pairs showed different aggression levels than other colony pairs, we did a matched pairs t-test on the proportion of aggressive interactions towards nestmates and non-nestmates to confirm the direction of behavioral trends. Each analysis was repeated separately for each of the three species combinations (conspecific for *Cr. levior*, conspecific for *Ca. femoratus* and heterospecific), and for the two categories of behavioral scoring (2–5, or 3–5 = aggression). We used R v 2.14.0 for all statistical analysis [46].

## Results

### Cuticular Hydrocarbons

**Figure 1:**
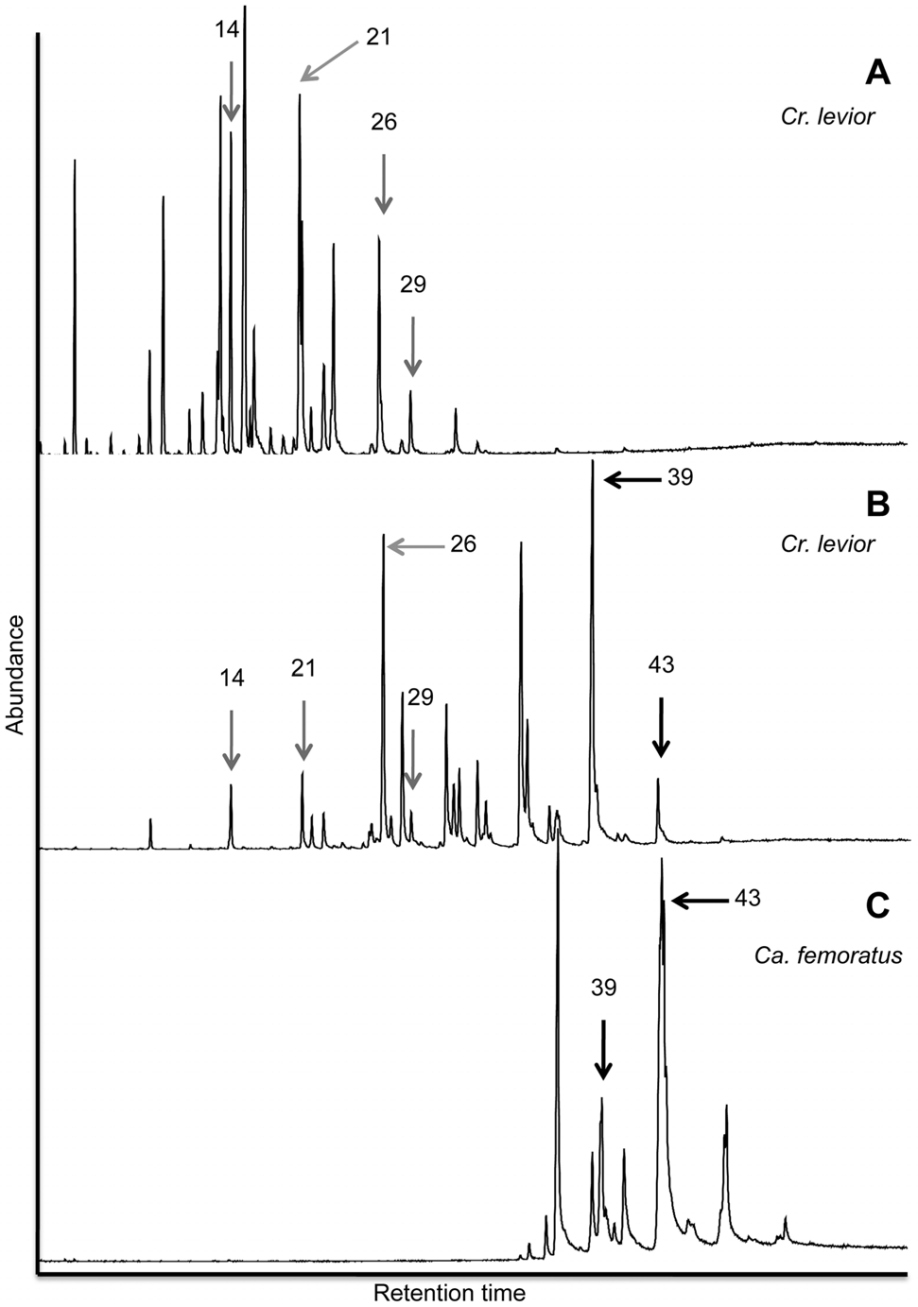
Representative chromatograms of the three chemotypes involved in the parabiotic nests. a) *Cr. levior* Type A, b) *Cr. levior* Type B, c) *Ca. femoratus*. Each peak represents a different hydrocarbon compound, as confirmed by spectral analysis. Compounds shared between species are shown by the arrows, with grey arrows showing peaks shared by only *Cr. levior* Type A and *Cr. levior* Type B, and black arrows being compounds shared between *Cr. levior* Type B and *Ca. femoratus*. Peak numbers refer to compound numbers in Table 2.

**Figure 2:**
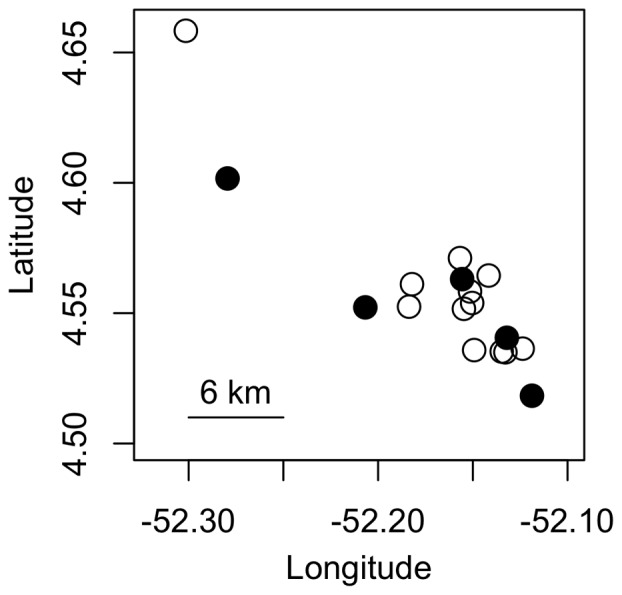
Map of nest locations showing 18 of the nests used in this study. Black circles represent *Cr. levior* Type A, and white circles represent *Cr. levior* Type B nests.

Surprisingly, we consistently recovered two distinct *Cr. levior* chemotypes, henceforth designated *Cr. levior* Type A and *Cr. levior* Type B (Fig. 1 a,b). Within each nest, however, there was only one *Cr. levior* chemotype (confirmed by analysis of individual chromatograms, data not shown). None of the nest cuticular hydrocarbon profiles appeared to be intermediate between *Cr. levior* Type A and *Cr. levior* Type B. Of the 20 colonies, 7 were of Type A, and 13 were of Type B. Ants from these two chemotypes were behaviorally and morphologically indistinguishable in the field. Examination by a taxonomic expert on *Crematogaster* who was blind to chemotype confirmed the lack of morphological differentiation between *Cr. levior* chemotypes (J. Longino, personal communication). The *Cr. levior* chemotypes overlapped in geographic distribution (Fig. 2), with one very distant nest (500 km away from main population, not shown in Fig. 2) sharing an almost identical CHC profile to *Cr. levior* Type B. No obvious topographical or landscape feature isolated the two chemotypes, and they appeared to occur sympatrically and sometimes very close together (<10 m between colonies of Type A and Type B, as verified by a sampling of other colonies not used in this study).

Across the three types of hydrocarbon profiles found in the parabiotic nests (two *Cr. levior* types and one *Ca. femoratus* type), there was a total of 78 different identifiable compounds, with some co-eluting for a total of 45 resolvable peaks. In general, *Ca. femoratus* compounds were of longer chain length than either *Cr. levior* type (Fig. 1c), and within the range observed previously for *Ca. femoratus* [40]. The profiles of *Ca. femoratus* and *Cr. levior* contained very few shared compounds (Table 2). Of the 45 peaks, only 2 compounds were shared amongst *Ca. femoratus* and *Cr. levior* Type B and no compounds were shared between *Ca. femoratus* and *Cr. levior* Type A. The two *Cr. levior* chemotypes shared only 4 compounds.

**Table 2:** Summary of average abundance of the 34 most abundant peaks from the pooled profiles of parabiotic ants.

#	Retention time (min)	Class of compound	Compound ID	*Ca. femoratus* (n = 20)	*Cr. levior* Type A(n = 7)	*Cr. levior* Type B (n = 13)
3	19.72	straight	C25	*trace*	9.7+/−6.4	
7	25.14	straight	C27		4.5+/−3.0	*trace*
8	26.09	single methyl	mix of 11me and 13me C27	*trace*	4.3+/−2.1	
12	28.94	single methyl	mix of 10me, 11me, 12me, 13me, 14me and 15me C28		1.2+/−0.5	
13	30.16	unsaturated	C29 alkene		10.8+/−4.4	
**14**	**30.98**	**straight**	**C29**		**6.4+/**−**2.0**	**4.0+/**−**1.4**
15	31.92	single methyl	mix of 7me, 9me, 11me, 13me, and15me C29	*trace*	23.1+/−6.7	*trace*
16	32.45	multimethyl	11,13 dime C29		1.2+/−2.7	
17	32.69	unsaturated	C30 alkene		1.4+/−2.1	
18	32.69	single methyl	5meC29		1.6+/−2.7	*trace*
19	32.75	multimethyl	11, 13 dime C30		1.2+/−2.0	
**21**	**36.06**	**unsaturated**	**C31 alkene**		**16.2+/**−**9.8**	**2.1+/**−**0.9**
22	36.82	straight	C31		*trace*	1.6+/−0.63
23	37.99	single methyl	mix of 7me, 9me, 11me, 13me, 15me, and 17me C31		5.3+/−4.7	*trace*
24	38.37	multimethyl	unidentified		3.7+/−2.1	
25	41.39	unsaturated	C33 diene			16.5+/−12.9
**26**	**41.98**	**unsaturated**	**C33 alkene**		**4.0+/**−**3.2**	**18.8+/**−**4.3**
27	42.56	straight	C33			1.6+/−1.3
28	43.37	single methyl	mix of 11me, 13me, 15me, and 17me C33	*trace*	*trace*	4.8+/−1.4
**29**	**44.03**	**multimethyl**	**unidentified**		**1.6+/**−**3.3**	**1.7+/**−**0.5**
30	47.14	unsaturated	C35 alkene and diene	*trace*	*trace*	21.2+/−5.5
31	48.88	single methyl	mix of 11me, 13me, 15me, and 17me C35	*trace*	*trace*	4.0+/−1.2
32	49.44	multimethyl	13,15,20,22 tetrame C34			1. 3+/−1.2
33	52.1	unsaturated	C37 diene	*trace*		8.1+/−6.2
34	52.82	unsaturated	C37 alkene	*trace*		3.0+/−2.7
35	53.97	single methyl	mix of 10me, 13me, 15me, 17me, and 19me C37	1.9+/−0.8		*trace*
38	54.78	multimethyl	13, 15 dime C38	18.1+/−4.7	*trace*	*trace*
**39**	**57.74**	**unsaturated**	**C39 alkene and diene**	**15.4+/**−**3.3**		**6.2+/**−**7.6**
40	58.91	single methyl	mix of 11me, 13me, 15me, 17me, and 19me C39	1.3+/−0.7		*trace*
41	59.6	multimethyl	unidentified	5.9+/−1.2		*trace*
42	61.72	straight	C40	1.7+/−4.8		
**43**	**62.51**	**unsaturated**	**C41 diene**	**41.8+/**−**6.8**	*trace*	**1.4+/**−**2.7**
44	66.2	unsaturated	C43 diene	1.8+/−2.7		*trace*
45	71.19	unsaturated	C45 diene	*trace*		

The percentages indicate the average relative proportion of each compound, as determined by the area under the peak in the chromatogram, +/− SD. The bolded compounds are highlighted in Figure 1. The word ‘trace’ indicates compounds only found in trace amounts (<1% of all profiles).

**Figure 3:**
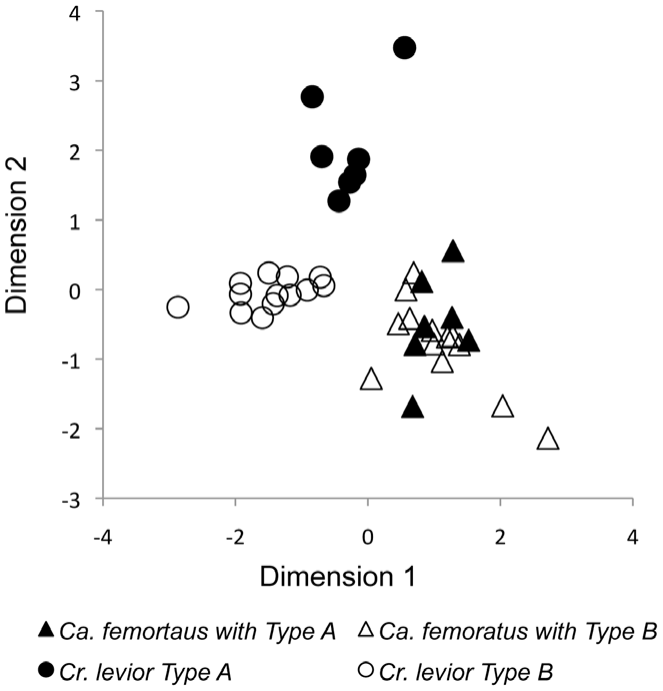
Nonmetric multidimensional scaling plot of the relative proportions of 45 cuticular hydrocarbon peaks from pooled ant profiles. Each shape represents the pooled profile of 30 *Cr. levior* or 5 *Ca. femoratus* worker ants of a different colony (n = 20 colonies).

When analyzed both qualitatively and quantitatively, the *Cr. levior* and *Ca. femoratus* profiles clustered separately from one another (Fig. 3). The *Cr. levior* Type A and *Cr. levior* Type B profiles were consistently different. In contrast, all *Ca. femoratus* possessed the same qualitative chemotype, regardless of whether they shared a nest with *Cr.* Type A or *Cr.* Type B (Fig. 1 c). This result was consistent when the analyses were repeated using only the *Ca. femoratus* profiles, and when including trace compounds (results not shown).

### Conspecific Recognition Behavior

In total, there were three between-type (*Cr. levior* Type A by *Cr. levior* Type B) colony pairs, two within-*Cr. levior* Type-A colony pairs, and five within-*Cr. levior* Type-B colony pairs. All colony pair comparisons were independent (ie: no colony was used twice). We were unaware of any chemotype differences at the time of the behavioral sampling, and only had colony pairings of all three combinations (axa, axb, bxb) by chance.

### Crematogaster Levior

**Figure 4:**
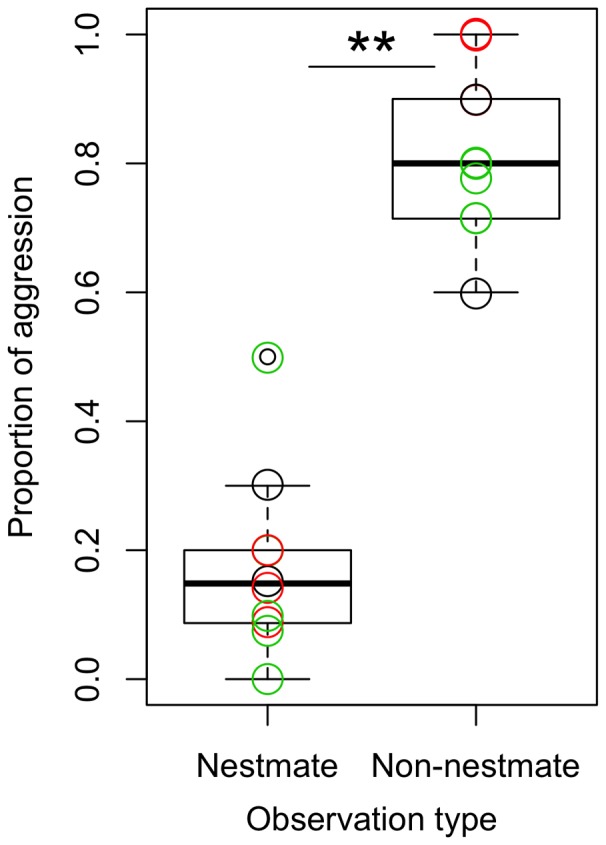
Proportion of aggressive behavior by *Cr. levior* in behavioral assays with nestmate and non-nestmate *Cr. levior* ants. The boxplot shows the mean +/− standard deviation. Black circles are for colony pairs considered within *Cr. levior* Type A combinations (n = 2), green circles are for within *Cr. levior* Type B combinations (n = 5), and red circles are for between *Cr. levior* Type A and *Cr. levior* Type B combinations (n = 3). The asterisks indicates there was significantly more aggression to non-nestmates (p<0.05).

There was a significant effect of observation category (whether nestmate or non-nestmate, χ_3,4_ = 60.2, p<0.001,), with colony pair and chemotype combination explaining 11.9% and 37.9% of the variance respectively. In all 10 of the nest combinations, *Cr. levior* ants displayed more aggression toward non-nestmates than toward nestmates (Fig. 4) (one tail paired t-test, t-ratio = 11.48, dF = 9, p = <0.01). This aggression was often typified by biting and fighting which often resulted in the death of one or both ants. This pattern of aggression was consistent whether the non-nestmate was of the same or of a different chemotype, but more aggression was displayed in pairings of non-nestmate ants of different chemotypes. Trophallaxis was rarely observed between non-nestmates (only 3/30 observed trophallaxes), and never between ants of the different chemotypes.

### Camponotus Femoratus

**Figure 5:**
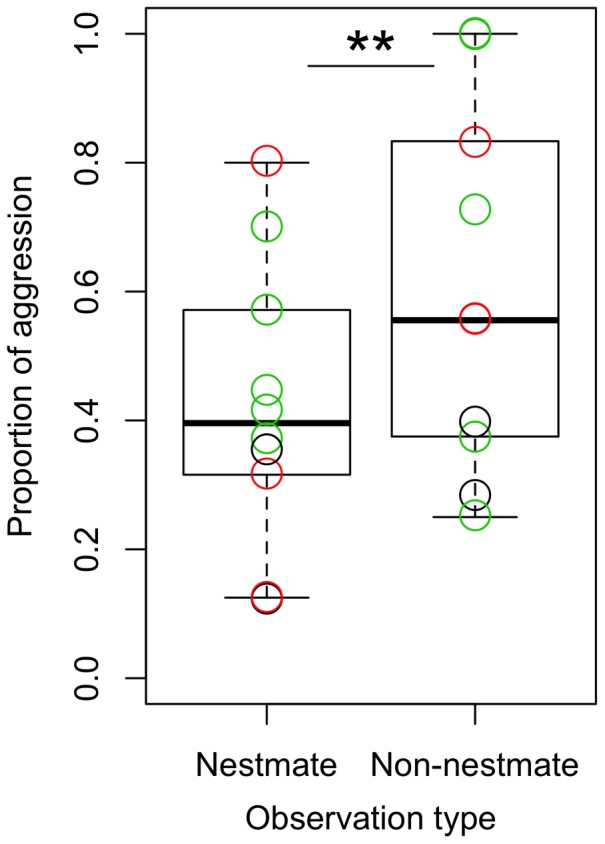
Proportion of aggressive behavior by *Ca. femoratus* in behavioral assays with nestmate and non-nestmate *Ca. femoratus* ants. Although *Ca. femoratus* was only of one chemotype, coloring is as in Figure 4 for consistency. The asterisks indicates there was significantly more aggression to non-nestmates (p<0.05).

There was a significant effect of observation category (whether nestmate or non-nestmate, χ_3,4_ = 4.2, p = 0.04), with no effect of chemotype combination (0% of variance), but with a significant effect of colony pair as a random effect (χ_3,4_ = 10.6, p = 0.001, 43.9% of the variance). Of the 10 nest combinations, only 7 displayed significantly more aggression toward non-nestmates than toward nestmates (Fig. 5), but there was an overall trend of more aggression toward non-nestmates (one tail paired t-test, t-ratio = −2.23, df = 9, p = 0.02). The conspecific *Ca. femoratus* aggression was less often fatal than conspecific *Cr. levior* comparisons, with ants often engaging in antennal boxing instead of direct biting and fighting conflicts. The boxing behavior was exclusively seen in the non-nestmate comparisons, and only in 3 of the 10 colony pairs, none of which were within-*Cr. levior* Type A comparisons. Aside from this occurrence of antennal boxing, there was no pattern related to the chemotype of the *Cr. levior* nesting partner (ie: *Ca. femoratus* is not more aggressive to non-nestmates that cohabitate with a different *Cr. levior* chemotype).

### Heterospecific Recognition Behavior

#### Cr. levior and Ca. femoratus

**Figure 6:**
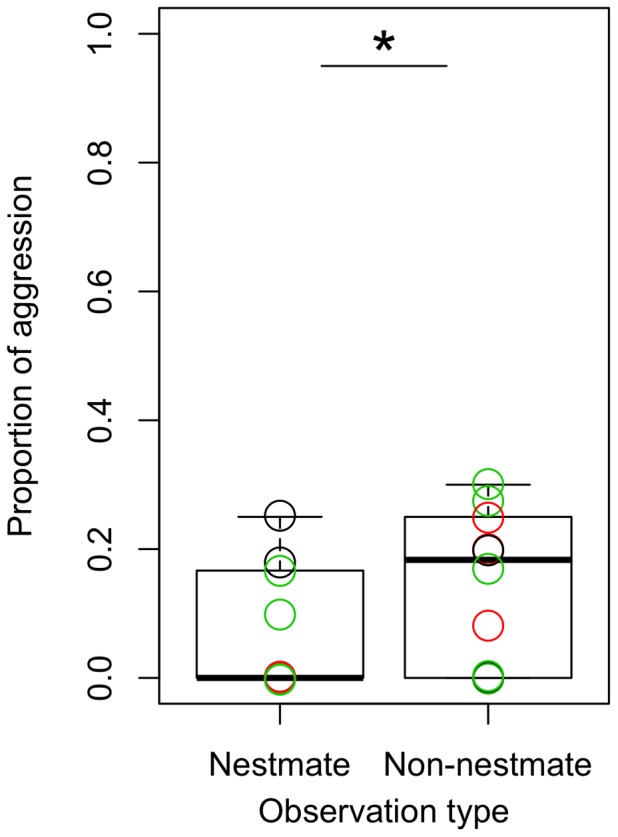
Proportion of aggressive behavior by *Cr. levior* in behavioral assays with nestmate and non-nestmate *Ca. femoratus* ants. Black circles are within *Cr. levior* Type B, green circles are within *Cr. levior* Type B, and red circles are for between *Cr. levior* Type A and *Cr. levior* Type B colony pairs. The asterisk indicates there was significantly more aggression to non-nestmates (p<0.10).

**Figure 7:**
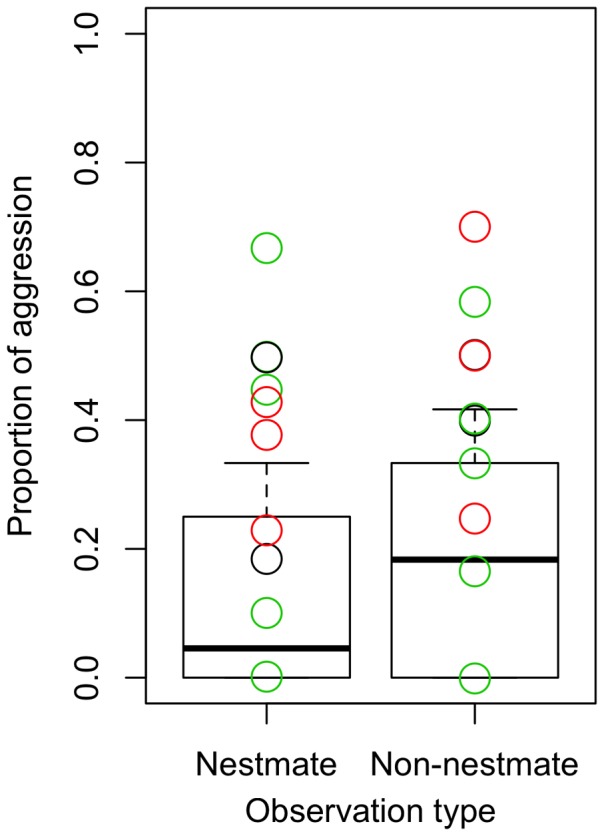
Proportion of aggressive behavior by *Ca. femoratus* in behavioral assays with nestmate and non-nestmate *Cr. levior* ants. Black circles are within *Cr. levior* Type B, green circles are within *Cr. levior* Type B, and red circles are for between *Cr. levior* Type A and *Cr. levior* Type B colony pairs. There was not a significant difference in aggression towards non-nestmates.

In general, there was less aggression observed in the heterospecific assays than in the conspecific assays. When aggression was analyzed without separating the behavior of the ants by species, there was no significant effect of observation category (nestmate or non-nestmate) (χ_3,4_ = 1.4, p = 0.24). We found that there was higher aggression displayed towards non-nestmates, but this effect was not significant at the 0.05 level for *Cr. levior* (χ_3,4_ = 3.1, p = 0.08 with 38.7% variance due to colony pair) or *Ca. femoratus* (χ_3,4_ = 1.1 p = 0.28, with 18.4% variance due to colony pair). Chemotype was not explanatory for either dataset (0% of variance). However, when considered significant at the 0.10 level, there was a difference in aggression of *Cr. levior*, especially when accounting for variation in colony pairs (Fig. 6, one tail paired t-test, t-ratio 1.77, df = 9, p = 0.06). For *Ca. femoratus*, this result was not significant (one tail paired t-test, t-ratio 0.26, df = 9, p = 0.40), but the trend was for increased aggression to non-nestmates (Fig. 7). This pattern was consistent regardless of whether the interaction was between or within chemotypes. In a few cases, extreme heterospecific aggression (resulting in the death of the *Cr. levior* ant) was observed, sometimes amongst nestmates. Heterospecific trophallaxis was only observed twice, with one occurrence between non-nestmates.

## Discussion

Ants typically have species-specific cuticular hydrocarbon profiles, with mostly quantitative differences between nests within a species. The surprising result of finding two very distinct *Cr. levior* chemotypes within parabiotic nests is unexpected because the two chemotypes were morphologically, behaviorally, and ecologically indistinguishable. It is highly probable that more cryptic types exist within the parabiotic *Crematogaster limata* complex [47], and we recommend using cuticular hydrocarbons as an informative phenotype to investigate possible cryptic differences within this group. Genetic analyses may provide insights into the extent of gene flow and genetic differentiation between chemotypes but, at present, we continue to regard both chemotypes as the species *Cr. levior*.

We found that *Cr. levior* and *Ca. femoratus* shared very few chemical cues, despite their nest-sharing lifestyle. This was also unexpected because other ants are known to actively acquire CHCs through social interactions with other ants [48], [49], as well as passively from the nesting material [50], physical contacts [51], and food sources [52]. This lack of chemical cue homogenization contrasts with the shared chemical cues in other multi-species social systems, such as socially parasitized mixed nests [2] and artificially mixed nests [26]. However, our results are consistent with findings from other socially symbiotic compound nests (see Table 1) [2], [23], [25], [36]–[39], [53], [54] in which the brood of the two species are kept physically separated, supporting the idea that mixed brood rearing facilitates chemical cue transfer. In artificially mixed nests, the degree of heterospecific chemical similarity scales with social interaction [27]. In these cases, ants only acquire heterospecific compounds through social interaction, and cannot synthesize hydrocarbons de-novo to match their heterospecific nestmates [55]. Given that the parabiotic ants in our study share nest space, immediate environmental conditions, and food sources, our findings suggest that non-environmental effects, such as social interaction, are required for chemical integration of social individuals.

Despite a lack of chemical cue homogenization, we found evidence that ants may recognize their heterospecific nestmates. Both species were more aggressive toward heterospecific non-nestmates than nestmates, with a more evident effect amongst *Cr. levior* ants. In NE Amazonia, recognition behavior has been studied in only one other parabiotic system: *Odontomachus mayi* and *Crematogaster limata parabiotica* [25]. These studies showed that ants attacked non-nestmates of the other parabiotic species, but tolerated heterospecific nestmates [25]. Our findings are consistent with this evidence, but we recommend caution before concluding that heterospecific nestmate recognition occurs amongst all socially symbiotic ants. In SE Asia, parabiotic ants could only distinguish amongst heterospecifics of common and foreign chemotypes [38], [44], [53], not specifically amongst nestmates. In all cases, some degree of heterospecific recognition seems to be a consistent difference between parabiotic and xenobiotic associations.

In the chemotype recognition of parabiotic ants of SE Asia, the dual chemotype species was the larger of the two ants (*Camponotus*) [38], in contrast to our system, in which the smaller *Crematogaster* has two chemotypes. Although we ensured in all observations that both species made antennal contact with the other, our assays highlight size-specific perceptual constraints because, despite being in close proximity to one another, *Ca. femoratus* (>1 cm in length) would frequently walk over its *Cr. levior* testing partner (2–3 mm) without hesitation. Indeed, size difference is a proposed mechanism for successful commensal compound nesting between *Pyramica* and *Platythyrea* [56]. Size differences have also been suggested as a mechanism to reduce foraging competition between the parabiotic species [34], [35]. The workers of the inquiline parasite *Acromyrmex insinuator* are also smaller than that of their sister-species host, which may help them escape heterospecific aggression [30]. Thus, there may be size-specific constraints on chemical cue perception, with size differences allowing the smaller *Cr. levior* to go undetected by the larger *Ca. femoratus*. This may explain why we found no significant evidence of heterospecific nestmate recognition by *Ca. femoratus*.

In our parabiotic system, and in previously studied parabiotic systems, the two species share few chemical cues but maintain some ability to recognize their heterospecific nestmates [25], [38], [44], [53]. Heterospecific recognition is consistent with the hypothesis that the recognition template used to assess nest-membership is learned and not self-referent, since it can expand to include another species phenotype [4]. Is there a cost to having an expanded recognition template? There is no evidence that either parabiotic species has lost the ability for conspecific recognition, which might happen if the recognition template was more generalized [4]. Both ant species involved in parabiotic social symbiosis maintain effective conspecific nestmate recognition behaviors, aggressively rejecting non-nestmates.

Ants distinguish amongst nestmates and non-nestmates by detecting both quantitative and qualitative differences in chemical phenotype [4], [12], [57], [58], but species are genetically constrained to produce only a limited range of compound classes and sizes [59]. The informational constraints on the chemical phenotype can be overcome by producing not only differing quantities of compounds, but also a broader range of compounds. We hypothesize that the long-chain unsaturated hydrocarbons of *Ca. femoratus*, found amongst several species of heterospecifically tolerated *Camponotus* ants [40], may be evolutionary novelties that facilitate heterospecific relationships, perhaps by opening new chemical information channels to communicate identity. Because both *Cr. levior* and *Ca. femoratus* were able to distinguish nestmates and non-nestmates of *Ca. femoratus* using only these unusual compounds, it is unlikely they are chemically insignificant or imperceptible [40]. The repeated evolution of these unusually long-chain alkenes and dienes suggest that they are a key trait that facilitates heterospecific tolerance [40].

In sum, we have found evidence that in parabiotic nests, 1) the recognition cues are not mimicked as in socially parasitized nests, but instead both species maintain a species-specific odor, 2) there is evidence of potential heterospecific nestmate recognition, and 3) conspecific recognition is maintained despite mutual heterospecific tolerance. The intact recognition systems in parabiotic social symbioses are distinct in many ways from the manipulated recognition systems in socially parasitized nests.

How is the cooperation of these social symbionts maintained in the face of potential exploiters? Cooperation is maintained through a combination of factors, such as compound nesting and novel chemicals, that minimize the heterospecific interference in nestmate recognition processes. In particular, the social symbiosis has likely been facilitated by each species using unique informational channels, by producing a different range of chemical cues and maintaining species-specific colony odors. This may be one reason that these social symbioses are so rare amongst social insects, and yet so common amongst *Camponotus* ants [33], [60] who have repeatedly evolved both heterospecific tolerance and unusual long-chain hydrocarbons [40]. Interference in the recognition system, a potential cost of living together, is minimized by such chemical innovations. There is certainly more work to be done investigating the frequency and distribution of such communication innovations, and their potential links to cooperative behavior. The maintenance of reliable recognition systems in these socially symbiotic nests supports the theory that parabioses are different from social parasitisms [32]. Our findings suggest that selection to maintain reliability in conspecific recognition can potentially constrain the evolution of interspecific cooperation.

## Supporting Information

Table S1 **Summary of GLMM results for nestmate and non-nestmate behavioral assays for the different pairings, with presence of aggression defined as behavioral scores 3–5.** The reported p-values are for comparisons between the full model, with observation category (whether nestmate or non-nestmate) as a fixed effect and chemotype combination and colony pair number as nested random effects, and the reduced model without observation category.
(DOC)
