# Supplementary material for: Recognition in a Social Symbiosis: Chemical Phenotypes and Nestmate Recognition Behaviors of Neotropical Parabiotic Ants
Source: PLoS One. 2013 Feb 22;8(2):e56492. doi: 10.1371/journal.pone.0056492 (PMC3579830; doi:10.1371/journal.pone.0056492)
Supplement: Table S1 — Summary of GLMM results for nestmate and non-nestmate behavioral assays for the different pairings, with presence of aggression defined as behavioral scores 3–5. The reported p-values are for comparisons between the full model, with observation category (whether nestmate or non-nestmate) as a fixed effect and chemotype combination and colony pair number as nested random effects, and the reduced model without observation category. (DOC) [file pone.0056492.s001.doc]

**Supplemental Table S1.** Summary of GLMM results for nestmate and non-nestmate behavioral assays for the different pairings, with presence of aggression defined as behavioral scores 3-5. The reported p-values are for comparisons between the full model, with observation category (whether nestmate or non-nestmate) as a fixed effect and chemotype combination and colony pair number as nested random effects, and the reduced model without observation category.

|  |  |  | % variance of random effects | | Fixed effects | | Significant difference | |
| --- | --- | --- | --- | --- | --- | --- | --- | --- |
| Species' behavior | Towards which species? | Distribution type | Colony pair | Chemotype combination | Chi-square, df=1 | p-value | p<0.05 | p<0.10 |
| **Conspecific comparisons** | | | | | | | | |
| *Cr. levior* | *Cr. levior* | Binomial | 29 | 30.2 | 54.016 | <0.01 | yes | yes |
| *Ca. femoratus* | *Ca. femoratus* | Binomial | 59.6 | 0 | 9.5521 | <0.01 | yes | yes |
| **Heterospecific comparisons** | | | | | | | | |
| unspecified | unspecified | Binomial | 38.6 | 0 | 1.6435 | 0.2 | no | no |
| *Cr. levior* | *Ca. femoratus* | Negative binomial | 19 | 0 | 2.3322 | 0.1 | no | yes |
| *Ca. femoratus* | *Cr. levior* | Negative binomial | 32.4 | 0.02 | 1.8802 | 0.2 | no | no |
